# PRMT3 and CARM1: Emerging Epigenetic Targets in Cancer

**DOI:** 10.1111/jcmm.70386

**Published:** 2025-02-18

**Authors:** Jiezuo Huang, Beining Qiao, Yixin Yuan, Yuxuan Xie, Xiaomeng Xia, Fenghe Li, Lei Wang

**Affiliations:** ^1^ College of Chinese Medicine Shandong University of Traditional Chinese Medicine Jinan China; ^2^ Xiangya College of Public Health Central South University Changsha China; ^3^ Hunan Normal University School of Medicine Changsha China; ^4^ Department of Gynaecology and Obstetrics, Second Xiangya Hospital Central South University Changsha China; ^5^ NHC Key Laboratory of Carcinogenesis and the Key Laboratory of Carcinogenesis and Cancer Invasion of the Chinese Ministry of Education, Cancer Research Institute, School of Basic Medical Science Central South University Changsha China

**Keywords:** cancer, CARM1, epigenetic, inhibitor, PRMT3

## Abstract

The family of protein arginine methyltransferases (PRMTs) occupies an important position in biology, especially during the initiation and development of cancer. PRMT3 and CARM1(also known as PRMT4), being type I protein arginine methyltransferases, are key in controlling tumour progression by catalysing the mono‐methylation and asymmetric di‐methylation of both histone and non‐histone substrates. This paper reviews the functions and potential therapeutic target value of PRMT3 and CARM1 in a variety of cancers. Studies have identified abnormal expressions of PRMT3 and CARM1 in several malignancies, closely linked to cancer progression, advancement, and resistance to treatment. Such as hepatocellular carcinoma, colorectal cancer, ovarian cancer, and endometrial cancer. These findings offer new strategies and directions for cancer treatment, especially in enhancing the effectiveness of conventional treatment methods.

## 1 Introduction

Epigenetics, a mechanism of gene expression regulation that does not involve changes in the DNA sequence, encompasses various processes including DNA methylation and histone modification [1, 2]. Protein arginine methylation stands out in these processes, notably the reactions catalysed by PRMT3 and PRMT4, playing crucial roles in a multitude of physiological and pathological processes [3, 4]. Recent studies indicate that PRMT3 and CARM1 play significant roles in the occurrence and progression of cancer. The protein arginine methylation in which they are involved is an important form of post‐translational modification that not only affects protein expression and activity but also participates in processes such as cell signalling, proliferation, apoptosis, and tumour invasion and metastasis. This makes them new potential targets for cancer treatment, offering us new therapeutic strategies.

The family of protein arginine methyltransferases (PRMTs) is ubiquitously present in mammals, becoming a new focal point in epigenetic studies. This family includes several members that influence key processes such as cell growth, differentiation, and proliferation through the catalysis of arginine methylation [5]. Numerous studies have shown the role of PRMTs in cancer, metabolism, neurodegenerative, and muscular diseases [6, 7, 8, 9, 10, 11]. Nine members of the PRMT family have been identified in mammals, where PRMTs transfer methyl groups from S‐adenosylmethionine to the guanidino nitrogen atoms on the arginine side chains of proteins, leading to the formation of methylated arginine, asymmetric dimethylarginine (ADMA), and symmetric dimethylarginine (SDMA). They are categorised into three types and nine varieties based on this function. Type I PRMTs (PRMT1, PRMT2, PRMT3, PRMT4 [also known as Coactivator‐Associated Arginine Methyltransferase 1, CARM1], PRMT6, and PRMT8) generate monomethylarginine and ADMA, Type II PRMTs (PRMT5 and PRMT9) generate monomethylarginine and SDMA, and Type III, PRMT7, exclusively produces MMA [5, 12, 13, 14, 15]. Members of the PRMT family possess highly homologous SAM‐dependent MTase catalytic domains, with a different motif structure outside of the MTase domain, these motifs can be called additional domains or motifs that not only increase the functional diversity of PRMTs but may also influence their substrate selectivity, intracellular localisation, and activity regulation, thus giving PRMTs important roles in diverse cellular processes including transcriptional regulation, RNA processing, and signal transduction [14]. The structures of PRMT family members are presented in Figure S1 of Supporting File 1. Members of the human PRMT1‐4 family share a conserved SAM‐dependent methyltransferase domain, including SAM and substrate binding pockets [16]. PRMT1 and PRMT6 contain only one MTase domain [17, 18]; PRMT2, PRMT3, PRMT4, PRMT5, PRMT8, and PRMT9 all have an N‐terminal motif preceding the catalytic domain [19, 20, 21, 22]. Although PRMT7 and PRMT9 both contain a repeated methyltransferase region, the C‐terminal domain in PRMT9 is referred to as a pseudo‐domain [23, 24]. The nine PRMT variants are encoded by distinct chromatin domains, overseeing a range of biological processes, including transcription regulation, RNA splicing, DNA repair, and ribosome homeostasis [25, 26, 27]. Each PRMT's function is distinctly dictated by its substrates [28]. The homo‐dimer or dimer‐like assembly of PRMTs refers to the complexes formed between these enzyme molecules through specific interactions, which are an important component of their biological functions. Understanding this assembly is crucial for elucidating the catalytic mechanisms and regulatory networks of PRMTs. Zhou et al. first explored the allosteric regulatory mechanism of PRMT1 dimerisation using a combination of molecular dynamics (MD) simulations, network topology analysis, and biochemical assays. Their study revealed that dimer formation can promote the binding of SAM and facilitate the catalytic methylation process, providing new directions for further allosteric studies within the PRMT family [29]. The Zhang, Zhou, and Cheng [30] demonstrated that the core region of PRMT3 forms dimers through crystal packing and solution behaviour experiments, a finding that provides important structural foundations for the functional characterisation of the PRMT family. These studies not only deepen our understanding of the structure and function of PRMTs but also provide theoretical support for developing therapeutic strategies targeting these enzymes. Furthermore, the PRMT family is continually expanding, with the recent discovery of new members PRMT10 and PRMT11. However, it should be noted that, so far, these two members exist only in plants. The existence of PRMT10 and PRMT11 in mammals and their potential functional roles remain unclear and require further exploration. In summary, these findings enrich our understanding of the diversity of the PRMT family and suggest that future research needs to explore the different functions of these enzymes in human diseases and their potential biological significance [31, 32].

**FIGURE 1:**
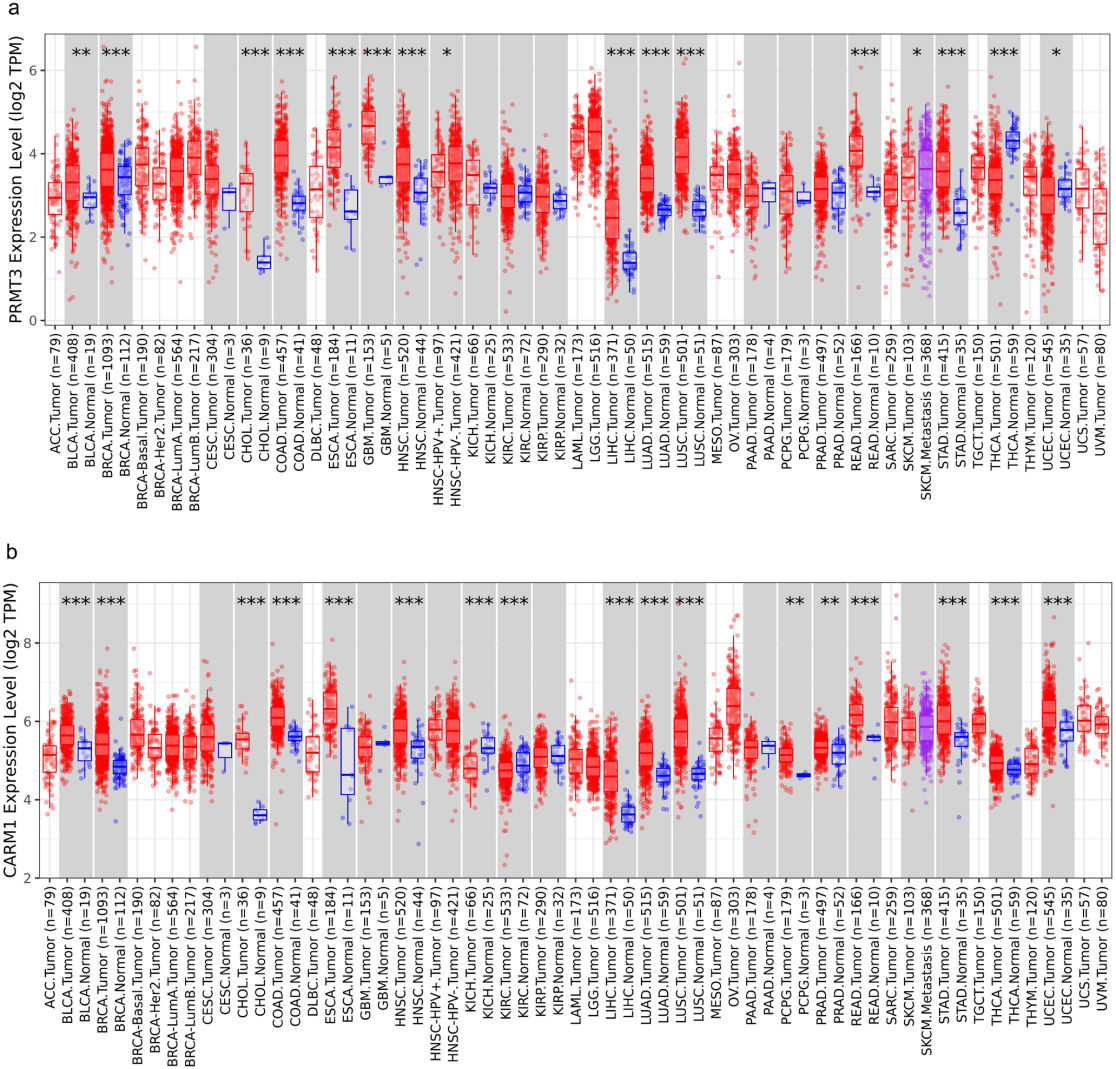
Differential expression of PRMT3 and PRMT4 (also known as Coactivator‐Associated Arginine Methyltransferase 1, CARM1) in different cancer (http://timer.cistrome.org/). (a) The expression of PRMT3 in different cancer of TCGA database is shown, where red represents the tumour group and blue represents the normal control group, as displayed in grey columns when normal data are available. (b) The expression of CARM1 in different cancer in TCGA database is shown, where red represents the tumour group and blue represents the normal control group, as displayed in grey columns when normal data are available. *p < 0.05; **p < 0.01; ***p < 0.001.

PRMTs play a key role in the development, progression, and treatment response of cancer. As a type I PRMT, PRMT1 has been shown to have an important role in a variety of cancers, such as bladder, ovarian, prostate, oesophageal squamous cell carcinoma, and acute myeloid leukaemia [33, 34, 35, 36, 37]. The growing body of research on PRMT1 has enhanced our understanding of its diverse substrates and its role in tumour biology, thus positioning PRMT1 as an intriguing target for new cancer therapy strategies [18]. Meanwhile, PRMT5, a significant member of type II PRMTs, has been the subject of widespread research. PRMT5 is key in the initiation and progression of cancer, with its regulatory roles and functions being critical to cancer development [38]. Inhibitors of PRMT5 have demonstrated effectiveness in treating solid and hematologic malignancies, especially likely to be more effective in cancers with splicing mutations [39]. A range of cancers display aberrant protein‐arginine methylation via type I PRMTs (primarily PRMT1 and PRMT4) and type II PRMTs (principally PRMT5). Research indicates that inhibition of type I and II PRMTs can reduce phosphorylation and overall ATR in cancer cells, presenting new approaches for cancer therapy [40]. Furthermore, as a member of the type I PRMT family, PRMT6 also plays a significant role in various cancers, including breast cancer, lung cancer, hepatocellular carcinoma, and pancreatic cancer. PRMT6 contributes to the initiation and progression of cancer by regulating gene expression, enhancing cell proliferation and migration, modulating signalling pathways, regulating cancer cell metabolism, and promoting the self‐renewal and differentiation of tumour stem cells [41]. In conclusion, the study of PRMTs in cancer offers new therapeutic strategies and directions, significantly impacting the development of more effective cancer treatments.

In the PRMT family, PRMT3 and PRMT4 function as type I enzymes and are central to numerous cellular processes by catalysing the asymmetric dimethylation of both histone and non‐histone substrates. Research indicates that the high expression of PRMT3 and PRMT4 in various malignancies is closely linked to the development and progression of cancer, as shown in Figure 1 (http://timer.cistrome.org/). By altering the acetylation and methylation patterns of histones and DNA, they remodel epigenetic alterations in tumour and immune cells, becoming promising targets in cancer research. Compared to other PRMTs such as PRMT1 and PRMT5, there are relatively fewer reviews available, hence this review focuses on the mechanisms of action of PRMT3 and PRMT4 in tumorigenesis, especially their roles in epigenetic modifications. Additionally, we discuss how the action mechanisms of PRMT3 and PRMT4 in tumours could inform future cancer drug development. As potential targets for cancer therapy, PRMT3 and PRMT4 have shown significant research and clinical value. Future research needs to further explore the specific mechanisms of these enzymes and overcome the limitations of current treatment strategies to develop more effective anti‐tumour drugs.

## 2 Structure and Function of PRMT3 and CARM1

**FIGURE 2:**
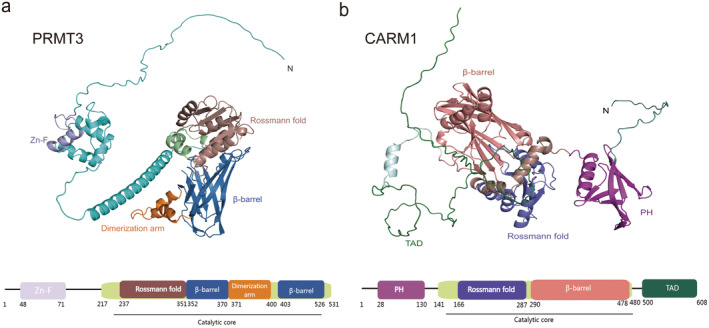
Structural features of PRMT3 and CARM1. (a) The domains and sub‐structures of a monomer of human PRMT3 (Alpha‐Fold, AF‐O60678‐F1). PRMT 3 consists of the N‐terminal domain, a C‐terminal domain, and a central PRMT catalytic core. The N‐terminal domain has a unique Zn‐F domain (lilac colour) that mediates interactions with various proteins. The central PRMT catalytic core consists of a Rossmann fold (brown) and two β‐barrels (blue). (b) The domains and sub‐structures of a monomer of human CARM1 (Alpha‐Fold, AF‐Q86X55‐F1). CARM1 is composed of an N‐terminal domain, a C‐terminal domain, and a central PRMT catalytic core. The N‐terminal domain has a pleckstrin homology (PH) (modena) domain and is involved in substrate recognition. The C‐terminal domain possesses a transcriptional activation domain (TAD) (bottle green) and is necessary for CARM1 to act as a transcriptional coactivator. The central PRMT catalytic core is composed of a Rossmann fold (indigo) and a β‐barrel (pink).

PRMT3, as a type I protein arginine methyltransferase, primarily catalyses the asymmetric dimethylation of arginine residues and plays a key role in cancer development through various mechanisms including cell metabolism and gene regulation. The substrates of PRMT3 encompass histones, RNA‐binding proteins, transcription factors, coactivators, and ribosomal proteins. The structural features of PRMT3 endow it with unique biological functions and regulatory mechanisms, as shown in Figure 2a (AF‐O60678‐F1‐v4). It contains a domain for binding S‐adenosylmethionine (SAM), with SAM acting as a methyl donor in the methylation reaction [30]. Differing from other PRMTs like PRMT1 and PRMT4, PRMT3's N‐terminus features a C2H2 zinc finger motif for substrate recognition, a structure thought to be closely linked to its substrate specificity and binding affinity [30]. Research by Bachand and Silver [19] has shown that the 40S ribosomal protein S2 is the first known physiological substrate of PRMT3. Moreover, the C‐terminal region of PRMT3 includes a catalytic domain accountable for its methyltransferase activity, associated with its substrate specificity and subcellular localisation [42]. The diverse substrates and unique structural features of PRMT3 provide key insights into its role in tumours. For example, methylation of HIF1α by PRMT3 could enhance angiogenesis and glycolysis, influencing colorectal cancer development; in glioblastoma, it might promote cell proliferation; and in breast cancer, assist in inducing apoptosis. C2H2 zinc fingers are a common protein structural domain that typically participates in DNA binding and transcriptional regulation. In PRMT3, this structure may influence its interaction with specific transcription factors, thereby regulating the expression of cancer‐related genes. Wang et al. defined the PRMT3‐IGF2BP1‐HEG1 axis as an important regulatory factor and therapeutic target for oxaliplatin resistance, indicating the potential of using PRMT3 expression levels as a biomarker for oxaliplatin resistance in HCC patients based on pre‐treatment biopsies [43]. Shi et al. [7] found that higher PRMT 3 expression levels were associated with reduced numbers of tumour‐infiltrating CD8+ T cells and poor response to ICB and identified PRMT 3 as a potential biomarker and therapeutic target to overcome immunotherapy resistance in HCC.

CARM1 functions in a range of biological processes such as cell differentiation, signal transduction, and regulation of gene transcription. CARM1 mainly acts on histones, specifically methylating the 17th and 26th arginine residues of histone H3 (H3R17 and H3R26), which is critical for gene expression regulation, especially during embryonic development and cell differentiation [44, 45, 46]. As the first transcriptional regulator identified in the PRMT family, PRMT4 can enhance the methylation of the p160 coactivator family (such as SRC‐1, SRC‐2/GRIP1, and SRC‐3/NCOA3/AIB1) and histone acetyltransferases (p300/CBP), thereby promoting gene activation [47, 48, 49, 50, 51]. PRMT4 can also methylate RNA‐binding proteins, such as HuR (Human antigen R) [52], which is significant for the stability and regulation of mRNA. The crystal structure analysis of CARM1 has uncovered its N‐terminal pleckstrin homology‐like domain (PH‐like), C‐terminal transactivase domain, and central catalytic domain, which includes four conserved PRMT motifs essential for binding to SAM (S‐adenosylmethionine) and arginine substrates [53, 54], as shown in Figure 2b (AF‐Q86X55‐F1‐v4). Notably, the N‐ and C‐terminal domains of CARM1 are not essential for its methyltransferase activity but are crucial for substrate recognition and transcription‐mediated activation [55, 56]. Recent studies have discovered new roles for CARM1 in mammalian development, cell differentiation, autophagy, and metabolism, as well as its involvement in pre‐mRNA splicing, mRNA retention, and stability of RNA regulation. With a deeper understanding of PRMT4 substrates and structural functions, the link between its aberrant activity and the development of various diseases (especially cancer) is becoming increasingly clear. Overexpression of CARM1 is closely associated with the occurrence of various cancers. Therefore, in‐depth studies on the structure and function of CARM1 and the development of effective CARM1 inhibitors are of significant importance for cancer prevention and treatment. Zhang et al. used biochemical and structural methods to specifically investigate the effect of acetylation at Lys(18) in the histone H3 tail peptide on CARM1 peptide recognition. Their findings provide important information for designing effective peptidomimetic inhibitors and offer new insights into the crosstalk mechanism between arginine methylation and lysine acetylation, laying the foundation for the development of CARM1 peptidomimetic inhibitors [57]. Van et al. [58] discovered that transition state simulations are a valuable tool for studying the structure of CARM1, aiding in the elucidation of the mechanisms by which the arginine methyltransferase family regulates arginine methylation in various cancers and other related diseases. The Boriack‐Sjodin team demonstrated the crystal structures of human CARM1 bound to three different peptide sequences: the S‐adenosylmethionine (SAM) analogue sinefungin, histone H3, and PABP1. This was the first time multiple motif sequences were resolved in a single PRMT enzyme, showcasing that the CARM1 binding site can accommodate various peptide sequences while maintaining the core binding mode for unmethylated and monomethylated substrates [59]. These studies not only deepen our understanding of the structure and function of CARM1 but also provide important theoretical support for developing therapeutic strategies targeting CARM1.

**FIGURE 3:**
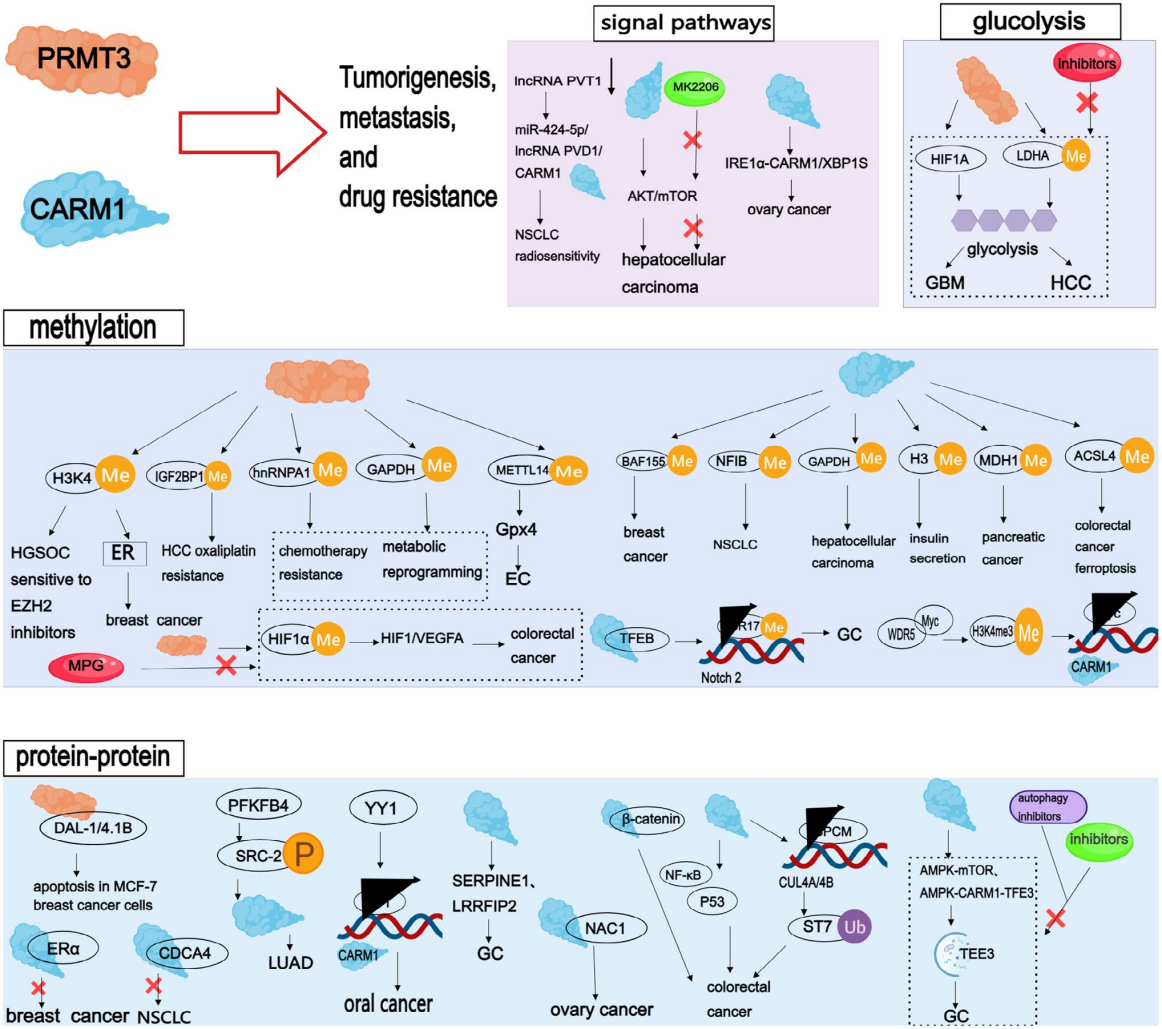
Mechanism of action of PRMT3 and CARM1 in different cancer. The mechanisms of action of PRMT3 and CARM1 in various cancers mainly involve arginine methylation, protein–protein interactions, signalling pathway regulation, and glycolysis regulation. Abnormalities in these mechanisms may lead to malignant biological behaviours such as proliferation, survival, and metastasis of cancer cells. In breast cancer, PRMT3 is involved in regulating H4R3me2a modification, thereby modulating the endoplasmic reticulum stress signalling pathway and promoting the proliferation and migration of cancer cells. In liver cancer, PRMT3 mediates the arginine methylation of IGF2BP1, increasing resistance to oxaliplatin. In pancreatic cancer, PRMT3 catalyses the methylation of hnRNPA1, increasing the expression of ABCG2 and thus enhancing chemotherapy resistance. Meanwhile, CARM1 catalyses the arginine methylation of MDH1R248, regulating the glutamine metabolism and redox balance of pancreatic cancer cells. In breast cancer, CARM1 interacts with BAF155 to drive tumour progression and metastasis. On the other hand, CARM1 activates the AKT/mTOR signalling pathway, promoting the progression of liver cancer. Additionally, in glioblastoma (GBM), PRMT3 enhances the expression and activity of HIF1A, thereby promoting glycolysis and exerting carcinogenic effects.

In addition to their well‐known protein arginine methyltransferase activity, PRMT3 and CARM1 also possess functions that do not depend on their enzymatic activity. Research from the Tsukada lab has indicated that CARM1 plays a non‐enzymatic role in splicing, suggesting that within the context of splicing, CARM1 functions as a scaffolding molecule rather than as an enzyme [60]. PRMT3 plays a significant role in gene regulation and various cellular functions, making it a therapeutic target for human cancers for a long time. Although some PRMT3 inhibitors have been developed to block the catalytic activity of PRMT3 [61], there has been limited success in removing PRMT3‐deposited ω‐NG and NG‐ADMA at the cellular level with small molecules. Moreover, the biological functions of PRMT3, particularly its non‐enzymatic functions in cancer cell lines, remain to be elucidated. The team led by Zhou hypothesised that merely inhibiting the enzymatic function of PRMT3 might not be sufficient to cause complete loss of function. To test this hypothesis, the team reported the development of a first‐in‐class PRMT3‐targeting PROTAC based on MDM2, which selectively reduces PRMT3 protein and ADMA levels. Unlike classical inhibitors, PROTACs have the advantage of modulating both enzymatic and non‐enzymatic protein functions simultaneously, thereby offering a potential strategy to overcome the limitations of inhibitors [62]. The findings of Dionne et al. indicate an intriguing cross‐regulatory pattern between the ZNF277–the 40S ribosomal protein uS5 (RPS2) and PRMT3‐RPS2 complexes. According to the analysis of available human tumours from the Human Protein Atlas project, overexpression of ZNF277 is associated with improved prognosis in human cancers [63]. In contrast, overexpression of PRMT3 reduces the amount of the ZNF277–uS5 complex, which is linked to poor prognosis in human cancers [63]. The opposing effects of ZNF277 and PRMT3 expression on human cancer prognosis suggest that targeting the ribosomal uS5 may reduce the growth of human cancers. In summary, PRMT3 and CARM1 are viewed as potential oncogenes, their overexpression significantly impacting a variety of cancers (as shown in Figure 3), such as colorectal, liver, pancreatic, prostate, endometrial, breast, [63] ovarian, oral cancers, and glioblastomas(as shown in Table 1).

**TABLE 1:** Multiple functions exerted by PRMT3 and CARM1 in various cancers.

	Cancer type	Express level	Related gens/proteins	Biological roles	Clinical significances	References
PRMT3	Glioblastoma	High	HIF1A	Promote the glycolytic metabolism	Promote GBM cell growth	[64]
	Oral cancer	High	YY1	Promote the YY1‐mediated activation of the reporter gene	Promote progression of oral cancer	[65]
	Breast cancer	—	DAL‐1/4.1B	Induction of apoptosis in MCF‐7 breast cancer cells	Regulator of apoptosis in adenocarcinoma cells	[66]
	Invasive micropapillary carcinoma	High	—	Promote H4R3me2a and regulation of the ER stress signalling pathway	Promote cells proliferation and metastasis; Poor clinical outcome	[67]
	Hepatocellular carcinoma	High	LDHA	Enhanced arginine methylation of LDHA	Promote glycolysis and HCC outgrowth	[68]
	Liver cancer	High	IGF2BP1	Promote IGF2BP1 stabilise HEG1 mRNA	Promote oxaliplatin resistance in HCC	[43]
	Pancreatic cancer	High	hnRNPA1, ABCG2	Methylate hnRNPA1 and increases the expression of ABCG2	Enhanced resistance to cancer chemotherapy	[69, 70]
		—	GAPDH	Methylate R248 position of GAPDH	Mediate metabolic reprogramming and cell proliferation	[71]
	Colorectal cancer	High	HIF1α, VEGFA	Stabilise HIF1α to regulate the HIF1/VeGFA signalling pathway	Promote colorectal cancer and correlate with overall survival	[72]
		High	C‐MYC	stabilise C‐MYC	Promote cells proliferation, migration, and invasion	[73]
	Prostatic cancer	high	—	—	—	[74]
	Endometrial cancer	—	METTL14	PRMT3 interacts with METTL14 and participates in its arginine methylation	Promote malignant progression and drug resistance	[75]
	—	—	RPS2	—	The prognosis of cancer is poor	[63]
PRMT4/ CARM1	Glioblastoma	High	WDR5, Myc	Promote Myc binding to the CARM 1 promoter	Promote cells proliferation and self‐renewal	[76]
		—	OCT4, SOX2, NANOG	—	—	[77, 78]
	Breast cancer	—	ERα, AIB1, E2F1	—	Stimulate breast cancer growth	[79]
		—	BAF155	—	Promote cancer progression and metastasis	[80]
	ER‐positive breast cancer	Positively correlated with ER α levels but negatively with tumour grade	ERα	A cofactor	Inhibit cell proliferation and induce differentiation	[81, 82]
	Non‐small‐cell lung cancer	—	miR‐424‐5p, lncRNA PVD1	—	Improve radiosensitivity of NSCLC	[83]
		—	CDCA4	Regulate autophagy	Inhibit EMT, migration, and invasion	[84]
	Lung adenocarcinoma	—	PFKFB4, SRC‐2	PFKFB4 enhance CARM 1 expression mediated by phosphorylation of SRC‐2	Promote cells proliferation, migration, and invasion	[85]
		—	circHMGB2, miR‐181a‐5p	—	Drive immunosuppression and anti‐PD‐1 resistance	[86]
	Small cell lung cancer	—	NFIB	CARM1 methylation	Promote rapid occurrence of the SCLC	[87]
	Hepatocellular carcinoma	—	GAPDH, AMPK	CARM1 methylates GAPDH at R234 and suppresses its activity in an AMPK‐dependent manner	Key regulatory mechanism of glucose metabolism	[88]
		—	AKT/mTOR	Activation AKT/mTOR signalling pathway	Promote HCC progression and predict poor prognosis	[89]
	Pancreatic cancer	Low	MDH1, ROS	Catalyse arginine methylation at R248 in MDH1	Regulate cells the glutamine metabolism and redox balance	[71]
	Gastric cancer	High	TFEB, Notch2, N2ICD, MAML1	Binding to TFEB and activate the transcription of Notch2; enhance binding N2ICD and MAML1	Promote cells proliferation and carcinogenesis	[90]
		High	AMPK‐mTOR, AMPK‐CARM1‐TFE3	Promote TFE3‐mediated autophagy	Promote GC progression	[91]
		—	SERPINE1, LRRFIP2 variant 3	Cooperative activation of the transcription of SERPINE1 with the LRRFIP2 variant 3	Promote invasion of gastric cancer cells	[92]
	Colorectal cancer	—	β‐catenin	Regulate β‐catenin‐mediated gene expression	Promote cells growth	[93]
		High	P53, NF‐κB	Regulate the transcription of P53 and NF‐ κB target genes	Regulate the biological functions of cancer cells	[94]
		High	miR‐195‐5p	—	Promote cells growth	[95]
		—	miR‐195	miR‐195 can inhibit CARM1	Enhance radiosensitivity of colorectal cancer cells	[96]
		—	CARM 1‐p300‐c‐Myc‐Max (CPCM) transcription complex, CUL4A/4B	CPCM binds to the promoter of CUL4/4B and causes ubiquitination and degradation of ST7	Promote the development of colorectal cancer	[97]
		—	RPF2, MYCN, ABCB1	RPF2 expression promotes binding of CARM1 to the nucleus and expression of ABCB1	Improve drug resistance	[98]
	Prostatic cancer	High	AR	—	Promote development of PCa	[94, 99]
		—	C3, AR	C3 treatment resulted in reduced binding of AR to target genes and reduced recruitment of AR and CARM1	Induce cell death	[100]
	Ovary cancer	—	NAC1	NACA and CARM1 Interaction	Promote development of ovary cancer and predict chemoresistance	[101]
		High	IRE1α/XBP1s	CARM1 regulates the expression of the XBP1s target genes	Promote development of ovary cancer	[10]
	Epithelial ovary cancer	High	EZH2	—	Promote development of epithelial ovarian cancer	[93]
	High‐grade serous ovarian cancer	High	SWI/SNF complex, EZH2/BAF155	Methylation of the SWI/SNF subunit BAF155 results in a switch of the promoter of the EZH2/BAF155 from BAF155 to EZH2	HGSOC selectively sensitive to EZH2 inhibitors	[80, 102, 103]
	HR‐proficient epithelial ovarian carcinoma	—	EZH2, MAD2L2	EZH2 inhibitor upregulated the MAD2L2	EOC sensitive to a poly PARP inhibitor	[104]
	Endometrial cancer	High	Selective inhibitor TP‐064	Induction of apoptosis	Inhibit cells proliferation	[105]
	Acute myeloblastic leukaemia	—	LYL1, AETFC	LYL1 recruits CARM1 to chromatin	Promote cells survival	[106]
	Multiple myeloma cells	High	p53	Activate p53‐signalling pathway	Inhibit cell proliferation and associate with a poor prognosis	[107]

## 3 Role of PRMT 3 and CARM 1 in Tumours

### 3.1 Glioblastoma

Glioblastoma (GBM) is the most common and aggressive primary brain tumour, with its growth and progression mechanisms not fully understood. The study by Liao's team discovered that PRMT3 is among the most significantly enriched members of the PRMT family in both high‐grade and low‐grade gliomas. Higher expression of PRMT3 is associated with poorer overall survival in glioma patients, and it has been confirmed that PRMT3 is crucial for the proliferation, survival, and tumour growth of GBM cells. Further research indicates that PRMT3 plays a key oncogenic role in GBM progression by enhancing the expression and activity of HIF1A, thereby promoting glycolytic metabolic programs [64]. Moreover, pharmacological targeting of PRMT3 can inhibit GBM cell growth by suppressing HIF1A expression and glycolysis, revealing PRMT3's key role in GBM growth and progression and suggesting it as a potential target for GBM therapy.

The study by Wang et al. [76] found that WD repeat domain 5 (WDR 5) promotes Myc lac 1 promoter by interacting with Myc and inducing histone 3 lysine 4 trimethylation (H3K4me3), thereby promoting proliferation and self‐renewal of glioblastoma and neuroblastoma cells. This was the first study to identify a gene or pathway regulating CARM1 expression, revealing that CARM1 is regulated by the WDR5‐Myc axis, providing key insights for the treatment of cancers with Myc overexpression. Additionally, Kappadakunnel's group found increased CARM1 expression in glioblastoma samples and associated changes in PRMT4 expression with poorer patient survival rates [108]. Previous studies have shown that CARM1 is associated with the regulation of OCT4, SOX2, and NANOG [77]. Álvaro and his team's research provided the first report on the increased gene expression profile of CARM1, detailing the embryonic stem cell gene expression characteristics of OCT4A, SOX2, and CARM1 genes in GBM samples. They found a significant increase in CARM1 expression, indicating that CARM1 is a promising therapeutic target [78].

### 3.2 Oral cancer

Behera's research identified that in oral cancer, CARM1 exhibits abnormally high expression levels and demonstrates carcinogenic functions. The study also revealed that YY1 acts as a positive regulator of the CARM1 gene promoter, and silencing YY1 in oral cancer cells significantly reduces CARM1 expression [65]. Furthermore, YY1 itself is a substrate for CARM1‐mediated arginine methylation, which can promote the activation of YY1‐mediated reporter genes in vivo. Therefore, a positive feedback loop is formed between CARM1 and YY1, mutually regulating each other, thereby driving the progression of oral cancer [65].

### 3.3 Breast cancer

Breast cancer is the most prevalent cancer in women, ranking first in incidence among all female malignancies, with cell metastasis being the leading cause of mortality [109]. Research by Jiang and Newsham [66] found that the tumour suppressor DAL‐1/4.1B can synergise with protein methylation to induce apoptosis in MCF‐7 breast cancer cells. Furthermore, PRMT3 was identified as an interacting protein of DAL‐1/4.1B. PRMTs perform post‐translational methylation of arginine residues in proteins, a modification associated with the regulation of various cellular processes including nucleocytoplasmic transport, signal transduction, and transcription [110]. This indicates that DAL‐1/4.1B and PRMT3 could be significant regulators of apoptosis in breast cancer cells [66]. In exploring the metabolic characteristics and related mechanisms of invasive micropapillary carcinoma (IMPC), Zhi et al. conducted targeted metabolomics analysis on tumours excised from patients with early‐stage IMPC (*n* = 25) and IDC (*n* = 26), and through integrating mass spectrometry, RNA sequencing, and ChIP‐sequencing data, they found overexpression of PRMT3 in IMPC, with high levels of PRMT3 associated with poor clinical prognosis. The research further proved that PRMT3 acts as a crucial regulator of breast cancer cell proliferation and metastasis, both in vitro and in vivo. Mechanistically, PRMT3 regulates the endoplasmic reticulum (ER) stress signalling pathway by promoting asymmetric dimethylation of histone H4 arginine 3 (H4R3me2a), endowing breast cancer cells with robust proliferative and metastatic capabilities [67]. Additionally, the study identified that small molecule inhibitors targeting PRMT3 activity could represent a promising method for breast cancer treatment, providing new avenues for the precise diagnosis and therapy of IMPC.

Among all cancer types, the role of CARM1 in ER‐positive breast cancer has been the most extensively studied. Frietze's early research identified CARM1 as a key factor in the oestrogen‐stimulated breast cancer growth pathway, downstream of ERα and AIB1 and upstream of the cell cycle regulatory transcription factor E2F1 [79]. Al‐Dhaheri's study established CARM1 as a coactivator capable of modulating the comprehensive regulatory process of genes through ERα, concurrently inhibiting cell proliferation and inducing differentiation. Furthermore, the study also found that in human breast tumours, CARM1 expression is positively correlated with ERα levels in ER‐positive tumours but negatively correlated with tumour grade, indicating that CARM1 is an important epigenetic target in ER‐positive breast cancer [81]. To further elucidate the function of CARM1 in breast cancer development, Wang and others used Zinc‐Finger Nuclease technology to knock out CARM1 from several breast cancer cell lines. They discovered that CARM1 facilitates tumour progression and metastasis through the methylation of the chromatin remodelling factor BAF155 [80]. However, the absence of extensive genomic and proteomic research to systematically uncover genomic binding sites, transcription targets, and cancer‐related substrates means the molecular mechanisms behind CARM1's regulation of oestrogen/ER‐mediated gene transcription activation are still not fully understood. Peng et al. focused on the high expression of CARM1 in ERα‐positive breast cancer and its association with poor prognosis. They found that CARM1 is primarily and specifically recruited to active enhancers bound by ERα and is crucial for the transcriptional activation of homologous oestrogen‐induced genes in response to oestrogen treatment. This indicates that CARM1 is essential for oestrogen/ERα‐induced transcriptional activation, breast cancer cell growth, and tumorigenesis, making CARM1 a potential drug target for ERα‐positive and endocrine‐resistant breast cancer [82].

### 3.4 Lung cancer

Karen's research group confirmed the critical role of CARM1 in lung development, noting that in the absence of CARM1, alveolar type II cells fail to complete differentiation and undergo excessive proliferation [111]. Wang et al.'s study revealed the impact of long non‐coding RNA (lncRNA) PVT1 on the radiosensitivity of non‐small cell lung cancer (NSCLC) through the miR‐424‐5p/lncRNA PVT1/CARM1 signalling pathway. They discovered that the knockdown of lncRNA PVT1 and CARM1, coupled with the overexpression of miR‐424‐5p, increased the radiosensitivity of NSCLC, offering new targets for NSCLC therapy [83]. Xu et al. [84] discovered that inhibition of CDCA4 induces epithelial‐mesenchymal transition (EMT), migration, and invasion in NSCLC cells, concurrently suppressing autophagy. Further, they confirmed that CDCA4 suppresses EMT, migration, and invasion in NSCLC by regulating autophagy through its interaction with CARM1, offering valuable insights for the early diagnosis, monitoring of progression, and prognosis assessment in NSCLC [84]. Meng, Chen, and Han [85] research showed that PFKFB4 promotes the proliferation, migration, and invasion of lung adenocarcinoma (LUAD) cells by enhancing the phosphorylation of SRC‐2‐mediated CARM1 expression, offering a new perspective for LUAD treatment. Zhang et al.'s [86] research demonstrated that the circular RNA circHMGB2 drives immunosuppression and anti‐PD‐1 resistance in lung adenocarcinoma and squamous cell carcinoma through the miR‐181a‐5p/CARM1 axis. Additionally, the combination of CARM1 inhibitor EZM2302 and anti‐PD‐1 antibody showed synergistic action in preclinical models, uncovering the crucial roles of circHMGB2 and CARM1 in the LUAD and LUSC TME, offering new approaches to boost the effectiveness of PD‐1 immunotherapy in LUAD or LUSC [86]. Gao et al. employed a small cell lung cancer (SCLC) mouse model to investigate the crucial role of CARM1 methylation of NFIB in its transformational activity. They found that CARM1 and the CARM1 methylation sites on NFIB are crucial for the rapid onset of SCLC and further confirmed that CARM1 methylation of NFIB promotes SCLC development, suggesting CARM1 as a potential therapeutic target for SCLC [87]. Wu et al. introduced a novel biomarker for NSCLC, specifically the aberrant PolyA selection of CARM1. They found that the proximal PolyA selection of CARM1, through loss of miRNA binding, upregulates CARM1's EMT inducers and releases miRNAs that downregulate the EMT inhibitor RBM47, serving as a pathological biomarker for mesenchymal tumours and tumour metastasis. Moreover, they observed that patients with shorter CARM1 3′‐UTR lengths have better responses to chemotherapy, particularly to cisplatin [112].

### 3.5 Liver cancer

Our research results indicate that PRMT3 has a novel oncogenic role in HCC by linking post‐translational modifications with cancer metabolism, making it a promising therapeutic target for HCC. Lei's research discovered significant upregulation of PRMT3 in hepatocellular carcinoma (HCC) and proved that PRMT3 facilitates glycolysis and HCC growth by enhancing arginine methylation of LDHA, serving as a potential biomarker for HCC patient prognosis [68]. Additionally, the study indicates that treatment with the PRMT3 inhibitor SGC707 effectively diminishes PRMT3‐induced glycolysis and tumour growth in HCC [68]. Oxaliplatin‐based chemotherapy is effective in treating HCC, but primary or acquired resistance to oxaliplatin remains a major clinical challenge. Shi's et al., utilising CRISPR/Cas9 activation libraries, transcriptome analysis, and both in vivo and in vitro functional validations, demonstrated that PRMT3‐mediated arginine methylation of IGF2BP1 enhances liver cancer's resistance to oxaliplatin. Mechanistically, PRMT3‐mediated oxaliplatin resistance is partly dependent on the methylation of Insulin‐like Growth Factor 2 mRNA Binding Protein 1(IGF2BP1) at the R452 site, which is crucial for the function of IGF2BP1 in stabilising HEG1 mRNA (an effector of the PRMT3‐IGF2BP1 axis) [43]. Therefore, the overexpression of PRMT3 could serve as a biomarker for oxaliplatin resistance in HCC patients.

Zhong et al. discovered that CARM1 methylates Glyceraldehyde‐3‐Phosphate Dehydrogenase (GAPDH) at the R234 site and inhibits its activity in an AMPK‐dependent manner. In hepatocellular carcinoma patients, the R234 site of GAPDH is in a low methylation state, with CARM1 levels positively correlating with the methylation degree at R234. This discovery suggests that CARM1‐mediated methylation of GAPDH is a crucial regulatory mechanism in the glucose metabolism of hepatocellular carcinoma [88]. The study by Du et al. [89] demonstrated that PRMT4 is overexpressed in HCC tumour tissues and facilitates the progression of hepatocellular carcinoma by activating the AKT/mTOR signalling pathway, also suggesting a poor prognosis. Functional research has indicated that the overexpression of PRMT4 promotes proliferation, migration, and invasion of HCC cells, whereas knocking down PRMT4 suppresses these malignant activities. Moreover, inhibiting AKT/mTOR signalling with MK2206 or rapamycin significantly mitigated the malignant phenotype mediated by PRMT4 [89]. Therefore, PRMT4 can serve as a valuable biomarker and potential therapeutic target for HCC.

### 3.6 Pancreatic cancer

Hsu's et al. [113] found that in pancreatic cancer cells tolerant to gemcitabine (GEM), the expression levels of PRMT3 and PRMT6 are significantly upregulated. They further investigated the molecular mechanism of PRMT3 in regulating gemcitabine resistance in pancreatic cancer, finding that PRMT3 enhances chemotherapy resistance by methylating heterogeneous nuclear ribonucleoprotein A1 (hnRNPA1) to increase the expression of ATP‐binding cassette transporter G2 (ABCG2) [69]. Later research showed that PRMT3 mediates metabolic reprogramming and cell proliferation by methylating the R248 site on the glycolytic enzyme GAPDH, indicating that dual blockade of GAPDH and mitochondrial respiration might offer a new approach to treat pancreatic cancer with overexpression of PRMT3 [70].

Kim et al. [114] discovered that CARM1 participates in insulin secretion by methylating histone H3 in pancreatic B cells, identifying CARM1 as a key factor in high glucose‐mediated histone methylation. Wang et al. [71] proved that the arginine methylation of malate dehydrogenase 1 (MDH1) at the R248 site, catalysed by CARM1, modulates glutamine metabolism and the redox balance in pancreatic cancer cells. Additionally, they discovered that CARM1's activity is suppressed by reactive oxygen species (ROS), indicating that CARM1 could serve as a ROS sensor, regulating MDH1 activity and glutamine metabolism in pancreatic ductal adenocarcinoma (PDAC) [71]. At the same time, they also observed that the expression of CARM1 in pancreatic cancer is significantly lower than in normal tissue, accompanied by the activation of MDH1, revealing the important role of CARM1 in regulating pancreatic cancer metabolism through MDH1 methylation.

### 3.7 Gastric cancer

Wang et al. [90] discovered that CARM1 is overexpressed in gastric cancer (GC) and correlates with adverse outcomes in gastric cancer patients. They demonstrated that CARM1 can bind to transcription factor EB (TFEB), inducing H3R17me2 methylation of the Notch2 promoter, thereby activating Notch2 transcription. Furthermore, CARM1 enhances the binding between N2ICD and MAML1 by methylating the Notch2 intracellular domain (N2ICD), thereby promoting the proliferation and tumorigenesis of gastric cancer cells [90]. Yang et al. observed upregulation of CARM1 in clinical GC tissues and cell lines, noting that higher CARM1 expression levels correlate with poorer prognosis. Additionally, it was shown that CARM1 promotes TFE3‐mediated autophagy and GC progression through the cytoplasmic AMPK‐mTOR and nuclear AMPK‐CARM1‐TFE3 signalling pathways [91]. More importantly, the study for the first time proved that CARM1 inhibitor treatment significantly inhibits GC tumour growth both in vitro and in vivo, and synergises with autophagy inhibitors, presenting a promising therapeutic strategy [91]. Lee et al. discovered that CARM1 co‐activates the transcription of SERPINE1 and variant 3 of LRRFIP2 in GC cells. Further research indicated that inhibition of CARM1 enzymatic activity leads to a decrease in SERPINE1 expression and subsequently eliminates the invasive potential of gastric cancer cells [92]. These findings reveal the multifaceted role of CARM1 in gastric cancer development and its potential as a therapeutic target.

### 3.8 Colorectal cancer

Zhang et al. reveal that in colorectal cancer, there is a significant elevation in PRMT3 expression levels, which correlates with patients' overall survival. Further analysis found that PRMT3 regulates the HIF1/VEGFA signalling pathway by stabilising HIF1α, particularly, PRMT3‐mediated methylation of HIF1αR282 plays a key role in the stabilisation of HIF1α [72]. These discoveries offer new pharmacological strategies to inhibit HIF1α expression, like MPG peptides, potentially serving as an effective treatment for colorectal cancer, particularly in patients resistant to anti‐angiogenic therapies. Hu's et al. [73] found that PRMT3 is overexpressed in colorectal cancer, promoting the proliferation, migration, and invasion of colorectal cancer cells, with the mechanism being the stabilisation of C‐MYC to promote tumorigenesis.

Ou et al.'s study indicates that CARM1 is a significant positive regulator of Wnt/β‐catenin transcription and tumour transformation. During colorectal cancer cell growth, CARM1 interacts with β‐catenin and positively regulates gene expression mediated by β‐catenin [93]. Through tissue microarrays, Kim's study reveals high expression of CARM1 in colorectal cancer and further uncovers that CARM1 significantly regulates the transcription of P53 and NF‐κB target genes, thus modulating cancer cell biological functions [94]. Zhang's group discovered an upregulation of CARM1 expression in colorectal cancer tissues, which promotes cell growth both in vitro and in vivo and is directly targeted by miR‐195‐5p. The identification of miR‐195‐5p and CARM1 as potential independent prognostic factors for colorectal cancer is a novel discovery [95]. Concurrently, Chen et al. [96] indicate that miR‐195 enhances the radiosensitivity of colorectal cancer cells through the inhibition of CARM1. Utilising mass spectrometry and immunoprecipitation, Lu et al.'s identified in colorectal cancer that c‐Myc, along with its partner MAX (Myc‐associated factor X), histone acetyltransferase p300, and CARM1, forms a CARM1‐p300‐c‐Myc‐Max (CPCM) transcription complex. This complex binds to the promoter of CUL4A/4B and induces its expression, activating the CUL4A/4B‐associated E3 ligase‐CRL4, which leads to the ubiquitination and degradation of ST7 by the CRL4 E3 ligase, promoting the occurrence of colorectal cancer [97]. Moreover, knocking out or inhibiting components of the CPCM significantly reduced tumour volume. Lu et al.'s research suggests that RPF2 may regulate the expression of ABCB1 in colorectal cancer through the CARM1‐MYCN pathway, thereby promoting resistance in colorectal cancer. Mechanistically, RPF2 expression facilitates the binding of the RPF2‐CARM1‐MYCN trio and CARM1's association with the nucleus, which in turn boosts the expression of the downstream protein ABCB1, enhancing colorectal cancer resistance [98]. Feng's research group has shown that inhibiting CARM1‐mediated methylation of ACSL4 can promote ferroptosis in colorectal cancer. From a mechanistic perspective, ACSL4 is methylated by CARM1 at arginine 339 (R339), thereby promoting the interaction between RNF25 and ACSL4, which further aids in the ubiquitination of ACSL4 [115].

### 3.9 Prostatic cancer

Analysing data from the TCGA and GEO databases, Grypari et al. discovered that in prostate cancer (PCa) tissues, compared to non‐tumour tissues, there is an upregulation of PRMT7 and PRMT3, and a downregulation of PRMT2 and JMJD6, indicating potential interactions between members of the PRMT family and PCa [74]. Kim et al.'s study indicates that the overexpression of CARM1 is associated with the development of PCa and its progression to androgen‐independent PCa. They discovered that the expression of androgen receptor (AR) target genes related to CARM1 could primarily result from non‐hormone‐dependent activity [94]. The androgen receptor (AR) is a major therapeutic target for aggressive prostate cancer. However, targeting the AR alone leads to drug resistance and disease relapse. Eugine Lee's research discovered a small molecule inhibitor (C3) of nuclear β‐catenin activity that inhibits the growth of prostate cancer cells by blocking the interaction between β‐catenin/T‐cell factor and β‐catenin/AR protein. Treatment with C3 results in diminished AR binding to target genes and reduced recruitment of AR and β‐catenin co‐factors, such as CARM1, thus triggering prostate cancer cell death [100]. Grypari's research found that PRMT1 and CARM1 are upregulated in the early stages of prostate cancer progression, with CARM1 further upregulated after treatment. This indicates that CARM1 could be one of the adaptation mechanisms for PCa cells in an androgen‐depleted environment. These findings suggest that the epigenetic network drives PCa progression by enhancing AR signalling, cell cycle, and epithelial‐to‐mesenchymal transition [99]. Through structure‐based virtual screening, Liang et al. identified seven compounds targeting CARM1 and HDAC2. Among these, CH‐1 had the strongest activity, demonstrating significant anti‐proliferative effects against various prostate‐related tumour cells [116]. The team successfully identified a novel dual‐target inhibitor of CARM1/HDAC2, potentially playing a crucial role in prostate cancer therapy.

### 3.10 Ovary cancer

Ovarian cancer is the most aggressive gynaecological malignancy worldwide, with its incidence significantly increasing over the past decade [117]. Nakayama's study identified the interaction between CARM1 and NAC1 in a protein complex, revealing a significant correlation between the expression levels of CARM1 and NAC1 [101]. This study suggests that high levels of NAC1 and CARM1 are associated with poor prognosis in ovarian cancer patients receiving adjuvant chemotherapy, providing a new direction for prognostic biomarkers predicting chemotherapy resistance in ovarian cancer. Furthermore, the study found that NAC1 is not only a transcriptional repressor [22] but may also act as a transcriptional activator, cooperating with its interacting partner CARM1 to exert carcinogenic potential in ovarian cancer cells [101]. Karakashev's research group found that EZH2 inhibitors are effective in treating epithelial ovarian cancer with high expression of CARM1 [102]. Fukumoto, Magno, and Zhang [103] team noted in their review that in high‐grade serous ovarian cancer (HGSOC), the SWI/SNF complex is regulated by CARM1. Mechanistically, CARM1‐mediated methylation of the SWI/SNF subunit BAF155 causes the promoter of the pro‐apoptotic gene EZH2/BAF155 to switch from BAF155 to EZH2, making HGSOC with upregulated CARM1 selectively sensitive to EZH2 inhibitors [80, 102, 103]. Following further investigation, the team found that EZH2 inhibitors increased the expression of MAD2L2 and rendered HR‐proficient epithelial ovarian cancer (EOC) sensitive to poly (adenosine diphosphate‐ribose) polymerase (PARP) inhibitors, dependent on CARM1. CARM1 promotes the silencing of MAD2L2 by methylating the BAF155 subunit of the SWI/SNF complex on the MAD2L2 promoter, facilitating the transition from the SWI/SNF complex to EZH2 [104]. These mechanisms and clinical insights offer new perspectives for the treatment of ovarian cancer. Karakashev's research group found that patients with high‐grade serous ovarian cancer (HGSOC) exhibiting high CARM1 expression have a poorer prognosis and limited treatment options. They introduced a novel strategy for treating ovarian cancer by targeting CARM1 with enhancer of zeste homologue 2 (EZH2) and ADP‐ribose polymerase (PARP) inhibitors. Notably, PARP inhibitors can enhance anti‐tumour immune responses, and this effect is not dependent on BRCA1/2 mutation status or homologous recombination (HR) deficiencies [118]. Lin et al. show that targeting the IRE1α/XBP1S pathway can effectively reduce CARM1 expression in ovarian cancer. In response to ER stress, CARM 1 regulates the expression of XBP1s target genes and directly interacts with XBP 1 s. In situ and patient‐derived xenograft models, inhibiting the IRE1α‐CARM1/XBP1S pathway effectively suppresses ovarian cancer both in vitro and in vivo [119]. In ovarian cancer models with immunologically active CARM1 expression, the IRE1α inhibitor B‐109 synergises with immune checkpoint blockade anti‐PD1 antibodies. Lombardi et al. demonstrate that CARM1 facilitates ovarian cancer growth by transcriptionally reprogramming fatty acid metabolism, leading to the production of monounsaturated fatty acids. They found that inhibiting the fatty acid desaturase SCD1 could serve as an effective treatment method for ovarian cancer with high CARM1 expression [120]. Thus, dietary or interventional strategies to reduce blood lipids and increase saturated fatty acid intake could further improve the anti‐tumour effectiveness of SCD1 inhibition‐based treatments for several cancers, including ovarian cancer, characterised by frequent overexpression and amplification of CARM1.

### 3.11 Endometrial cancer

Wang et al.'s research found that PRMT3‐mediated methylation of arginine in METTL14 promotes the malignant progression and drug resistance of endometrial cancer (EC). Further experiments showed that inhibiting PRMT3 can enhance the sensitivity of EC cells to ferroptosis [75]. Mechanistically, PRMT3 interacts with METTL14 and participates in its arginine methylation. The absence of PRMT3 leads to the inability to bind and methylate METTL14, resulting in the upregulation and downregulation of Gpx4 in an m6A‐dependent manner, ultimately suppressing the cell's resistance to ferroptosis and sensitising EC to treatment. It is noteworthy that blocking PRMT3 can improve the suppressive impact of cisplatin and radiation therapy on endometrial cancer [75]. Inoue et al. found an increased expression of CARM1 in endometrial cancer. They discovered that the selective inhibitor of CARM1, TP‐064, can inhibit the proliferation of endometrial cancer cells by inducing apoptosis [105]. These discoveries offer new targets and strategies for treating endometrial cancer, particularly in terms of enhancing the efficacy of conventional treatment approaches.

### 3.12 Blood system tumour

Chen et al. uncovered the mechanism by which the oncogene LYL1 functions in acute myeloid leukaemia (AML). They found that LYL1 promotes the survival of AML cells by recruiting CARM1 to chromatin, playing a crucial role in AETFC assembly and gene activation. These findings not only offer new insights into gene activation mediated by AETFC but also further underscore the significance of CARM1 as a potential therapeutic target for AML [106]. On the other hand, research by Yang's group indicates that CARM1 inhibits the proliferation of multiple myeloma (MM) cells by activating the p53 signalling pathway. They noted high expression of CARM1 in patients with multiple myeloma, especially in those with stage III or relapsed/refractory MM, which is closely linked to poor prognosis. Further experiments showed that combining effective CARM1‐targeting shRNA with bortezomib significantly inhibits MM cells [107]. These discoveries provide crucial evidence for clarifying the pathogenesis of MM and identifying potential therapeutic targets, and they open up new avenues for the potential use of CARM1 in the treatment of MM.

### 3.13 Other diseases

Zhu et al. show that PRMT3 is upregulated in zebrafish in response to viral infection, and that overexpression of PRMT3 suppresses the cellular antiviral response. The PRMT3 inhibitor SGC707 enhanced antiviral capabilities. Additionally, PRMT3 is believed to regulate antiviral innate immunity through the catalysis of arginine methylation in the 5′‐triphosphate ribonucleic acid sensor rig [121]. Subsequent research, Zhu et al. [122] discovered that PRMT3 is a negative regulator of cytoplasmic RNA and DNA sensors. Moreover, the use of PRMT3 inhibitors made mice more resistant to RNA and DNA viral infections, further proving the regulatory role of PRMT3 on cytoplasmic RNA and DNA sensors, as well as its importance in antiviral innate immunity [122]. Besides its role in immune regulation, PRMT3 has also shown significant functions in other areas. It has been discovered that PRMT3 acts as a co‐activator for the liver X receptor, regulating the synthesis of liver triglycerides, and that pharmacological inhibition of PRMT3 exhibits potential in treating liver steatosis and lowering lipid levels [123]. Verma's research group demonstrated through yeast two‐hybrid screening and other experimental validations that PRMT3's interacting partner ALDH1A1 and its regulated retinoic acid signalling pathway are associated with disease [124]. Further studies indicate that PRMT3 regulates protein function not only by adding methylation marks but also through protein–protein interactions to modulate gene expression, affecting diverse biological processes [124]. The function of PRMT3 in chronic kidney disease (CKD) has also been a focus of interest. Research has found that PRMT3 can enhance CKD‐induced vascular calcification by promoting the glycolysis process following the methylation of arginine in HIF‐1α, thereby facilitating the occurrence of vascular calcification. These research findings provide crucial insights into the mechanisms of action of PRMT3 in various diseases and offer a new perspective on PRMT3 as a potential therapeutic target [125].

Yan et al.'s [126] discovered that CARM1 facilitates angiogenesis by interacting with YB1 and upregulating VEGF, indicating CARM1 as a potential therapeutic target for ischemic angiogenesis. Hartsough's group discovered that CARM1 binds to VEGFR‐2 through its highly conserved EVH1 and PH domain‐like N‐terminal domain, mediates methylation on R817 of VEGFR‐2, promotes tyrosine phosphorylation of VEGFR‐2, and regulates filopodia [127], which is significant for the development of new VEGFR‐2 inhibitors. Wang et al. [128] discovered that CARM1 aggravates doxorubicin (DOX)‐induced cardiomyopathy by facilitating ferroptosis via the inhibition of the Nrf2/GPX4 pathway, finding that is significant for deciphering the molecular mechanism of DOX‐induced ferroptosis. Clemons' research indicates that CARM1 can regulate nitric oxide synthase uncoupling and cerebral blood flow in Alzheimer's disease patients, with its activity significantly affecting CBF and NOS function in AD pathology [129]. Zhong et al.'s [130] demonstrated that CARM1 methylation of peroxisome proliferator‐activated receptor‐gamma (PPARg) at Arg240 enhances its interaction with PR domain‐containing protein 16 (PRDM16), promoting adipose tissue browning and thermogenesis. Lai et al. [131] discovered that endotoxin increases cellular concentration of CARM1 by reducing the levels of SCFBXO9, leading to lung epithelial cell death, suggesting that CARM1‐mediated lung epithelial cell death may play a significant role in endotoxin‐induced damage. Garbutt et al. [132] demonstrate that CARM1 significantly regulates cardiomyocyte maturation and is linked to the pathogenesis of human heart disease. Stouth et al. [133] showed that CARM1 regulates mitochondrial autophagy, autophagy, and atrophy processes, suggesting that targeting this enzyme could offer new therapeutic approaches for alleviating skeletal muscle atrophy. Zhang et al. [134] unveiled CARM1's role in regulating glucose metabolism in osteoblasts and osteoclasts, thereby affecting bone cell differentiation, providing new perspectives for treating conditions such as osteoporosis. Lim et al. [135] demonstrated that cisplatin‐induced ototoxicity is mediated by PRMT3, CARM1, and the endocannabinoid system, where PRMT3 functions by interacting with FAAH1 in HEI‐OC1 cells, indicating that targeting PRMT3 and CARM1 might be an effective strategy for treating ototoxicity and mitigating cisplatin side effects.

## 4 Inhibitors of PRMT3 and CARM1

Protein arginine methyltransferases (PRMTs) represent a crucial aspect of epigenetics, playing a role in the epigenetic regulation of cell destiny and being involved in numerous signalling pathways associated with tumour initiation, progression, and invasion. Arginine methylation occupies a pivotal role in the epigenetic regulation of disease development and progression, providing new pathways for the discovery and treatment of cancer medications. Numerous studies have shown that PRMT3 and CARM1 are overexpressed or dysregulated in cancer, hence the development of potent and selective inhibitors against PRMT3 and CARM1 has attracted widespread attention. However, due to the complexity of arginine methylation, the mechanisms behind the anticipated therapeutic benefits of PRMT inhibitors are not yet fully understood. Currently, numerous studies are dedicated to developing PRMT inhibitors for the treatment of malignant tumours. Based on their mechanism of action, PRMT inhibitors are categorised into competitive and non‐competitive inhibitors. Competitive inhibitors, such as AMI‐1 (selectively targeting PRMT1), bind competitively to the SAM‐dependent MTase catalytic domain of the corresponding PRMT, competing with the protein substrate; Non‐competitive inhibitors, such as MS023 (acting on type I PRMTs), do not inhibit competitively with the substrate but bind to pockets on the substrate protein to exert their inhibitory effect [136, 137]. Interestingly, SAM binding competitive inhibitors may non‐specifically bind to other types of methyltransferases, such as DNA methyltransferases. This non‐specific binding not only diminishes the therapeutic efficacy of the inhibitors but also potentially increases the risk of side effects. For example, if a PRMT inhibitor simultaneously affects DNA methyltransferases, its potential side effects could include adverse impacts on cellular proliferation, differentiation, and repair mechanisms, leading to a series of negative reactions. Therefore, the developed inhibitors must not only possess specificity for PRMTs but also need to have selectivity for each member of the PRMT family. The necessity for this specificity and selectivity is reflected in the functional differences among the various members of the PRMT family. For instance, members such as PRMT1, PRMT3, and PRMT5 play distinct roles in cellular proliferation, differentiation, and apoptosis. If an inhibitor lacks selectivity for a specific PRMT, it may affect multiple targets, resulting in unexpected biological effects or toxicity. For example, if an inhibitor targeting PRMT1 also inhibits PRMT5, it could disrupt normal immune responses or cell cycle control, which not only weakens the anti‐tumour effect but might also lead to serious side effects in patients. When developing new PRMT inhibitors, researchers should prioritise these characteristics to maximise therapeutic efficacy and minimise potential side effects. To this end, the design of inhibitors should consider the optimisation of their small molecular structures, enabling them to selectively bind to specific PRMTs and exert their effects without cross‐reacting with other methyltransferases. Furthermore, the assessment of the selectivity of PRMT inhibitors should include both in vitro and in vivo experiments. These experiments will help researchers understand the actions of the inhibitors in complex biological environments, including their pharmacokinetics, pharmacodynamics, and safety profiles. Specifically, in vitro experiments can be used for preliminary screening of the inhibitors' specificity by measuring their inhibitory effects on various PRMTs at different concentrations to evaluate selectivity. In vivo experiments, on the other hand, can provide more realistic physiological context data to observe the overall impact of the inhibitors on the organism. This comprehensive evaluation process will help lay the foundation for clinical applications and promote the development of therapeutic strategies targeting PRMT‐related diseases. As our understanding of the roles of PRMTs in various biological processes deepens, the newly developed targeted drugs are expected to play significant roles in the treatment of tumours, inflammation, and other diseases associated with PRMT.

Currently, inhibitors targeting PRMT3 and CARM1 are actively being developed to inhibit the activity of these enzymes, (as shown in Table 2), thereby disrupting the survival and proliferation of tumour cells. The development of these inhibitors is grounded in a thorough understanding of PRMT3 and CARM1's mechanisms of action within tumours and an extensive analysis of their expression and functionality across different cancer types. S‐adenosylhomocysteine (SAH) is a lead compound for constructing potent methyltransferase inhibitors. However, most SAH‐based PRMT inhibitors only contain 5′‐thioadenosine or its analogues and guanidino functional groups, losing interaction with the homocysteine binding site. Thus, the development of homocysteine analogues is crucial for enhancing the inhibitory activity of SAH analogues. Currently, two cases have been reported: one is the CARM1 inhibitor SKI‐73(6a), and the other is a dual‐substrate inhibitor of CARM1. The team led by Cai demonstrated that SKI‐73 (referred to as 6a in this work) exhibits drug‐like properties as a CARM1 chemical probe, capable of rapidly penetrating the cell membrane and subsequently being processed into an active inhibitor, retaining a tenfold enrichment within cells for several days. The potency, selectivity, mechanism of action, and target binding rate of these compounds were thoroughly characterised. Additionally, SKI‐73 (6a) summarised the impact of CARM1 knockout on the invasion of breast cancer cells [138]. Deng et al. [139], starting with the PRMT dual‐substrate inhibitor II710 as a lead compound and through structural modifications and structure–activity relationship (SAR) studies, discovered that analogues created by replacing guanidino with ureido showed enhanced PRMT3 inhibitory activity. When the substituent is a benzyl group (YD1113), the SAH analogue exhibits the strongest inhibitory activity against PRMT3/4/5. Furthermore, YD1113 exhibits substrate competitiveness only against SAM, differing in its inhibitory mechanism from dual‐substrate inhibitors [139]. In addition, the study by Halby et al. found that a crystal structure showed the compound could bind to the active site of CARM1, strongly displacing the S‐adenosyl‐l‐methionine cofactor and occupying its binding site. The compound interacts with the arginine substrate site through its cytidine component, and these findings open new avenues for the design of novel and effective CARM1 inhibitors based on the 5‐methylcytidine‐adenosine scaffold [140]. Professor Wu independently developed a small molecule inhibitor for type I PRMTs, CTS2190, exhibiting strong enzymatic inhibition against PRMT1/3/4/6/8 and specificity for type I PRMTs, while being highly selective against other epigenetic targets and kinases. The development of this inhibitor aims to meet the clinical treatment needs of NSCLC, pancreatic cancer, or other solid tumours, filling a gap in this therapeutic area domestically. Drew identified the CARM1 inhibitor EZM2302 (GSK3359088) with potent in vitro activity in preclinical models of aggressive myeloma, and treatment with EZM2302 in MM cell lines inhibited PABP1 and SMB methylation and cellular arrest [141]. Iannelli et al. transformed a non‐selective inhibitor of type I protein arginine methyltransferases into an effective and selective inhibitor of protein arginine methyltransferase 4 using a combination of deconstruction‐reconstruction and fragment growth methods. Despite low cell permeability, they observed a significant reduction in arginine methylation levels and a marked decrease in proliferation in MCF7 cells. They also reported crystal structures supporting the observed specificity and selectivity across various PRMTs [142]. The study of protein methylation is still at an early stage, with the binding sites and mechanisms of action for most drugs related to PRMTs remaining undefined. Furthermore, there is still a lack of research on PRMT inhibitors at the cellular level and in vivo. Hence, there is a need for more comprehensive research into the role of protein methylation modifications in the development of tumours to identify more effective and selective PRMT inhibitors.

**TABLE 2:** The related inhibitors of PRMT3 and CARM1.

Name of drug/inhibitor	Chemical formula	Target	IC_50_ (nm)	KDa	PDB ID	References
PRMT3‐allosteric inhibitor	—	PRMT3	50	38.62	3SMQ	[143]
SGC707	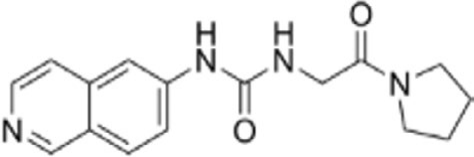	PRMT3	31	0.29834	4RYL	[68, 121]
SKI‐73	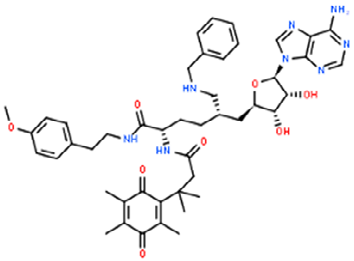	CARM1	1.1 ± 0.1μm	0.8504	—	[138]
EPZ0025654 (GSK3536023)	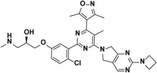	CARM1	3	0.57708	—	[142]
EZM2302 (GSK3359088)	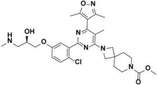	CARM1	6	0.58009	—	[142]
TP‐064	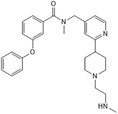	CARM1	< 10	0.4586	5U4X	[105, 144]
UM079	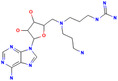	PRMT1/CARM1	—		6S7C	[145]
UM249	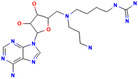		—	161.84	6S7B	
AA175	—		—	162.48	6S7A	
AA183	—		—	162.28	6S79	
UM305	—		—	162.2	6S74	
WH5C	—		—	162.72	6S71	
UM251	—		—	162.17	6S70	
MS023	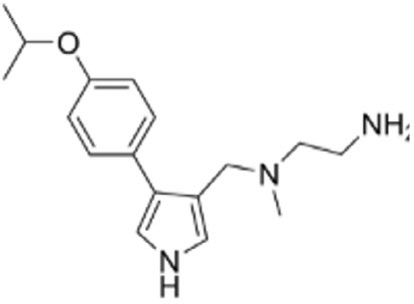	1/3/4/6/8	30/119/83/4 /5	0.2874	—	[132, 146]
YD1113	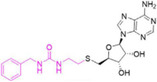	3/4/5	< 5 nmol/L	41.61	8G2H	[125]
CTS2190	—	PRMT1/3/4/6/8	—	—	—	—

*Note:* The symbol “—” represents: Not applicable.

## 5 Summary and Perspective

Considering the multifaceted roles of PRMT3 and CARM1 in tumour cell processes such as transcription, splicing, signal transduction, and DNA damage response, predicting the outcomes of PRMT3 and CARM1 inhibitors is challenging. Moreover, the interactions between PRMT3 and CARM1 with other proteins mean that inhibiting one PRMT might significantly impact the activity of another, complicating the treatment approach. Therefore, clarifying the molecular and biological functions of the complexes formed by PRMT3 and CARM1 interactions with proteins in different tumour cells is crucial for understanding the anti‐tumour activity of PRMT3 and CARM1 inhibitors. In considering PRMT3 and CARM1 as therapeutic targets, it is also necessary to consider their essential functions in development and normal cell homeostasis. At present, research on the regulation of PRMT enzymes at both transcriptional and post‐transcriptional levels is focused on a limited array of systems. For instance, post‐translational modifications (such as methylation, phosphorylation, and ubiquitination) have been found to regulate the activity, function, and stability of PRMT and its protein–protein interactions, necessitating future research on other post‐translational modifications and their mediating enzymes as potential therapeutic targets [147]. Another consideration is the occurrence of mutations in arginine residues within histones in specific cancer types. These mutations might possess oncogenic functions akin to those of carcinogenic lysine mutations within histones [148]. A systematic analysis of how these mutations impact neighbouring histone modifications, PRMT activity, and the overall gene expression profile should offer a robust strategy for creating personalised medications for treating tumours harbouring these specific mutations. Given the distinct arginine methylation profiles of individual tumours, the synergistic effects of these drugs may vary across different cancers. Therefore, further in‐depth research is needed to provide more precise treatments for combination use in cancers with specific genetic backgrounds. Besides cancer cells, PRMT can also regulate immune responses, including T cell function, anti‐tumour immunity, and cytokine signalling. This opens avenues for targeting these immunooncological mechanisms. Notably, a recent study on spliceosome‐targeted therapy showed that disruption of mRNA splicing promotes intrinsic antiviral signalling, downstream adaptive immune responses, and cell death in cancer cells [149]. Considering the crucial role of PRMT in splicing regulation, exploring whether PRMT inhibitors can employ a similar mechanism and if combining PRMT inhibitors with immunotherapy offers potential therapeutic advantages is a worthwhile direction. Although there has been significant advancement in this area, numerous challenges persist, such as enhancing the specificity and effectiveness of inhibitors and minimising their adverse impacts on healthy cells. Furthermore, due to the heterogeneity and complexity of tumours, developing personalised treatment strategies for specific tumour types or patient groups is also an important direction for future research.

The functions of PRMT3 and CARM1 in the onset and development of tumours, as well as the creation of inhibitors aimed at these enzymes, have emerged as crucial areas of study within cancer epigenetics. These studies are not only hot topics in molecular biology and oncology but also important targets for the development of anti‐tumour drugs. In conclusion, the potential of PRMT3 and CARM1 in cancer therapy is increasingly being revealed, with their inhibitors anticipated to become significant instruments in anti‐tumour treatment. With further research into the roles of these enzymes in tumours, we look forward to the development of more effective and safer treatment methods in the future.

## Author Contributions

**Jiezuo Huang:** writing – original draft (equal). **Beining Qiao:** data curation (equal), investigation (equal), writing – review and editing (equal). **Yixin Yuan:** data curation (equal), investigation (equal), writing – review and editing (equal). **Yuxuan Xie:** data curation (equal), investigation (equal), writing – review and editing (equal). **Xiaomeng Xia:** investigation (equal), writing – review and editing (equal). **Fenghe Li:** conceptualization (equal), supervision (equal). **Lei Wang:** conceptualization (equal), supervision (equal).

## Conflicts of Interest

The authors declare no conflicts of interest.

## Supporting information


Figure S1.

## Data Availability

Data sharing is not applicable to this article as no datasets were generated or analysed during the current study.
