# Supplementary figures and images for: PRMT3 and CARM1: Emerging Epigenetic Targets in Cancer
Source: J Cell Mol Med. 2025 Feb 18;29(4):e70386. doi: 10.1111/jcmm.70386 (PMC11834966; doi:10.1111/jcmm.70386)

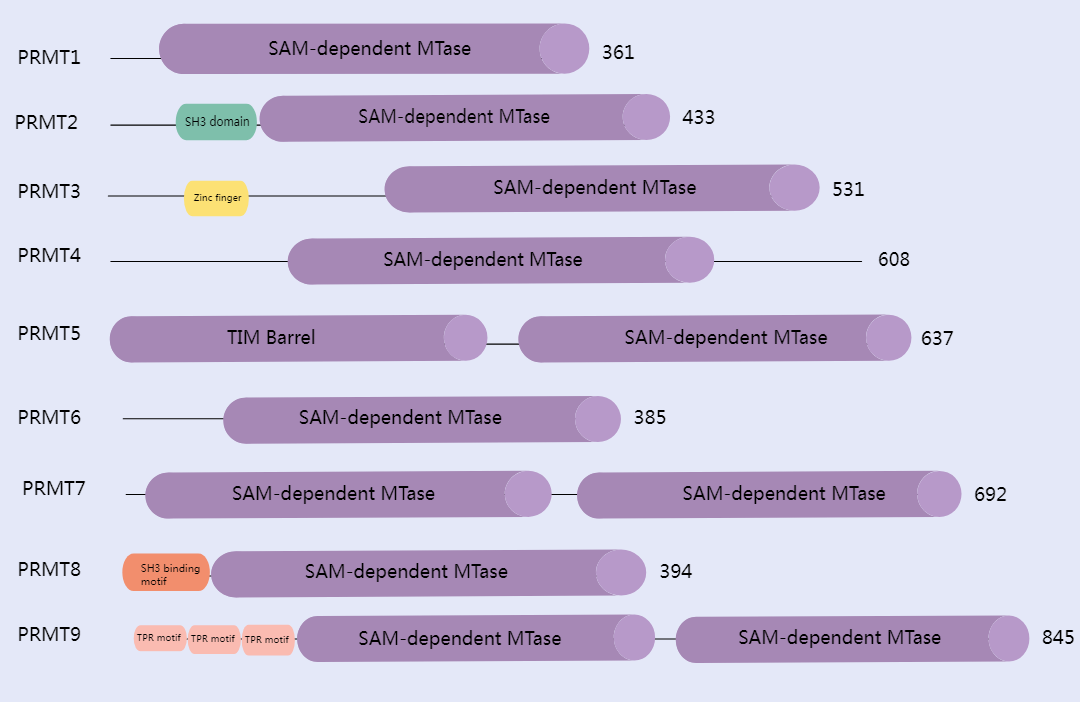

Supplement: Supplementary file 1 — Figure S1. [file JCMM-29-e70386-s001.tiff]
